# *Gata4* regulates fibroblast migration in a fibroblast-source-dependent manner under physiological matrix stiffness

**DOI:** 10.3389/fcvm.2026.1865878

**Published:** 2026-09-16

**Authors:** Zhentao Zhang, Jethro Wang Zih-Shuo, Peng Chen, Yanping Xu, Zhiyu Yan, Qingning Zhang, Xi Wang, Usman Alizai, Takahide Ikeda, Bryan A. Whitson, Timothy M. Pawlik, Hua Zhu

**Affiliations:** Department of Surgery, Davis Heart and Lung Research Institute, The Ohio State University, Columbus, OH, United States

**Keywords:** cardiac fibroblast, fibroblast migration, GATA4, mechanobiology, substrate stiffness

## Abstract

Fibroblast migration contributes to the spatial organization of tissue repair and extracellular matrix remodeling, but the transcriptional mechanisms regulating this process under physiologically relevant mechanical conditions remain incompletely understood. We investigated whether GATA4 regulates fibroblast migration in a tissue-of-origin-dependent manner. Cardiac fibroblasts (CFs), lung fibroblasts (LFs), and tail-tip fibroblasts (TTFs) were isolated from neonatal male and female *Gata4^fl/fl^* mice and cultured on 8-kPa polydimethylsiloxane substrates. CF behavior on 8-kPa substrates was first compared with that on conventional tissue-culture plastic. *Gata4* was subsequently deleted by adenoviral Cre transduction, and multiple features of steady-state migration were quantified by live-cell imaging. CFs cultured on 8-kPa substrates exhibited a smaller cell area and greater migration speed, displacement, and directional persistence than cells cultured on plastic, establishing a physiologically relevant mechanical baseline. Baseline *Gata4* expression was highest in CFs, intermediate in LFs, and lowest in TTFs, whereas adenoviral Cre reduced *Gata4* expression by more than 90% in all three populations. *Gata4* deletion produced distinct migratory responses according to fibroblast source. In CFs, *Gata4* loss reduced instantaneous speed, line speed, segment length, and directional persistence while increasing cell area. LFs showed modest increases in instantaneous speed, path speed, normalized 1-h path distance, and segment length without a clear change in directional persistence, whereas TTF migration was comparatively insensitive to *Gata4* loss. Integrated effect-size analysis confirmed that the coordinated alteration of migration rate and directionality was most pronounced in CFs. RNA-seq analysis of adult CFs further showed enrichment of general motility and actin-remodeling programs following *Gata4* deletion, without coordinated enrichment of lamellipodium and focal-adhesion programs. Together, these findings indicate that GATA4 supports efficient and persistent fibroblast migration in a fibroblast-source-dependent manner under physiologically relevant mechanical conditions.

## Introduction

Cardiac fibrosis is a common pathological response to myocardial injury and chronic stress and is characterized by excessive accumulation of extracellular matrix (ECM) proteins within the myocardial interstitium (1, 2). This remodeling process contributes to ventricular stiffening, impaired diastolic filling, and progression to heart failure (3). Cardiac fibroblasts (CFs) are the primary source of structural collagens in the heart and play a central role in both physiological matrix maintenance and pathological fibrotic remodeling (4, 5). Under homeostatic conditions, resident CFs, marked by expression of transcription factor 21 (Tcf21), maintain a relatively quiescent phenotype and support the structural integrity of the myocardial interstitium (6). In response to injury or sustained mechanical stress, CFs become activated, expend, and redistribute within the damaged myocardium (7–10) Their intrinsic migratory capacity influences how fibroblasts move through and spatially organize within the evolving injury environment. Efficient movement requires coordinated actin remodeling, protrusion formation, adhesion turnover, and cellular contractility (11–13) Although the mechanisms controlling myofibroblast activation and matrix production have been extensively studied (7, 8), the transcriptional mechanisms that regulate CF migration remain poorly defined.

The mechanical environment is a major determinant of fibroblast morphology and migration. Reported myocardial stiffness estimates depend on the measurement method. The Young's modulus of diastolic adult human myocardium has been summarized as approximately 8–15 kPa, and cardiac magnetic resonance elastography measured values of 7.2–11.8 kPa, with a median of 8.2 kPa, in healthy volunteers (14, 15).Conventional tissue culture plastic has a modulus on the order of gigapascals—several orders of magnitude stiffer. This supraphysiological stiffness promotes cell spreading, *α*-smooth muscle actin incorporation into stress fibers, and focal-adhesion maturation (16, 17) independently of exogenous stimulation (18, 19). These cytoskeletal and adhesive changes can also alter the balance between cellular contractility and translocation (20), complicating the interpretation of experiments performed on rigid culture surfaces. Polydimethylsiloxane (PDMS) substrates tuned to 8 kPa have been validated as an effective platform for maintaining CFs in a more physiologically relevant, quiescent state *in vitro* (18, 21). Culturing on these substrates limits stiffness-induced activation and preserves fibroblast morphology compared with conventional plastic (21, 22). This system therefore provides an appropriate baseline for distinguishing fibroblast-intrinsic regulation of migration from changes imposed by the culture substrate.

GATA4 is a zinc-finger transcription factor with well-established roles in cardiac development and cardiomyocyte (CM) biology, including regulation of the hypertrophic response, sarcomeric gene expression, and CM survival following pressure overload (23–25) During embryogenesis, GATA4 contributes to proepicardial formation and the specification of cardiac progenitor lineages that give rise to resident CFs in the adult heart (26). In CM, cardiac-specific *Gata4* deletion causes progressive ventricular dysfunction, an impaired hypertrophic response, and increased apoptosis under pressure overload (27, 28). More recently, conditional deletion of *Gata4* in Tcf21-lineage CFs disrupted CF homeostasis and worsened cardiac outcomes following myocardial infarction (29). These findings support a fibroblast-autonomous role for GATA4 but do not define whether GATA4 directly regulates the cytoskeletal and migratory behavior of CFs. Moreover, fibroblasts derived from different tissues retain distinct transcriptional and mechanosensory properties. Whether GATA4 regulates migration through a broadly conserved fibroblast mechanism or has a preferential role in the cardiac lineage therefore remains unknown.

In the present study, we tested the hypothesis that GATA4 supports efficient fibroblast migration under physiologically relevant matrix stiffness and that this function depends on tissue of origin. We first compared the morphology and intrinsic migratory capacity of primary CFs cultured on 8-kPa PDMS substrates or conventional tissue-culture plastic. We then deleted *Gata4* in CFs, lung fibroblasts (LFs), and tail-tip fibroblasts (TTFs) isolated from *Gata4^fl/fl^* mice and quantified multiple features of steady-state migration by live-cell imaging. Selected ECM-related transcripts were examined to determine whether the migratory response occurred alongside changes in matrix-remodeling gene expression. Finally, RNA-seq analysis of adult CFs following *Gata4* deletion was used to evaluate associated motility, actin-cytoskeletal, and cell-adhesion programs. This design allowed us to distinguish shared and cardiac-enriched responses to *Gata4* loss and to relate the observed migratory phenotypes to associated transcriptional changes.

## Materials and methods

### Animals

All animal experiments were conducted in accordance with the NIH Guide for the Care and Use of Laboratory Animals and were approved by the Institutional Animal Care and Use Committee (IACUC) of The Ohio State University. *Gata4^fl/fl^* mice (STOCK *Gata4*tm1.1Sad/J; JAX Stock #008194), in which loxP sites flank exons 3–5 of the *Gata4* locus, were obtained from The Jackson Laboratory and maintained on a mixed genetic background. Neonatal mice (postnatal day 0–3) were used for fibroblast isolation. Both sexes were included.

### Cell isolation

Primary CFs, LFs, and TTFs were isolated from neonatal *Gata4^fl/fl^* mice (postnatal day 0–3). All cells were cultured on gelatin-coated 8 kPa PDMS substrates in DMEM (Gibco, cat# 11965118) supplemented with 10% FBS (Sigma-Aldrich, cat# F2442) and 1% penicillin–streptomycin (Gibco, cat# 15-140-163) at 37°C in a humidified atmosphere of 5% CO₂. Cardiac fibroblasts were isolated using the Neomyts Neonatal Cardiomyocyte Isolation Kit (Cellutron, cat# nc-6031) following the manufacturer's protocol. Dissociated cardiac cells were seeded onto gelatin-coated 8 kPa PDMS dishes and allowed to attach for 40 min, after which non-adherent cardiomyocytes were removed by aspiration. LFs and TTFs were isolated by tissue explant. All three fibroblast populations were used at passage 0.

### PDMS substrate preparation

8-kPa PDMS substrates were prepared as previously described (30) with minor modifications. Briefly, Sylgard 184 (base and curing agent at 10:1 w/w) and Sylgard 527 (Part A and Part B at 1:1 w/w) were prepared separately and combined at a ratio of 0.102:9.90 (w/w). The mixture was vortexed, centrifuged at 3,200 × g for 3 min, and 500 µL was pipetted onto each 35 mm dish. Dishes were degassed under vacuum for 30–45 min and cured overnight at 50°C. Cured PDMS surfaces were sterilized with 70% ethanol under UV for 20–30 min, then coated with 0.1% gelatin solution (STEMCELL Technologies, cat# NC1620050) for 1 h at 37°C.

### Adenoviral transduction

GATA4 deletion was achieved by transduction with Ad-Cre-GFP adenovirus (University of Iowa Viral Vector Core). Ad-GFP was used as a control. Adenovirus was added at MOI 100 for 12 h, after which media was replaced. Experiments were performed 48 h post-transduction. Transduction efficiency was verified by GFP fluorescence (≥90%) and by measurement of *Gata4* mRNA levels by qRT-PCR.

### Immunofluorescence

Cells were fixed in 4% paraformaldehyde for 15 min, permeabilized with 0.05% Triton X-100 for 5 min, and blocked with Universal Blocking Buffer (BiogeneX) for 15 min. Primary antibodies: anti-*α*-SMA (Sigma, cat# A2547, 1:500) and anti-Vimentin (Novus Biologicals, cat# NB300-223, 1:500). Secondary antibodies: Donkey anti-Mouse IgG Alexa Fluor 555 (Thermo Fisher, cat# A-31570, 1:500) and Donkey anti-Chicken IgY Alexa Fluor 647 (Thermo Fisher, cat# A78952, 1:500). Cells were mounted with Fluoroshield Mounting Medium with DAPI (Abcam, cat# 502585553). Images were acquired on a Nikon A1 confocal microscope. Secondary-only controls showed no detectable fluorescence in the corresponding channels (data not shown). Enlarged views in Figure 1 were obtained from representative regions of the original whole-dish images. Cytoskeletal activation was identified by increased *α*-SMA intensity, stress-fiber-like organization, and increased cell spreading. Vimentin was used to visualize the intermediate-filament cytoskeleton and was not used alone as an activation marker.

**Figure 1:**
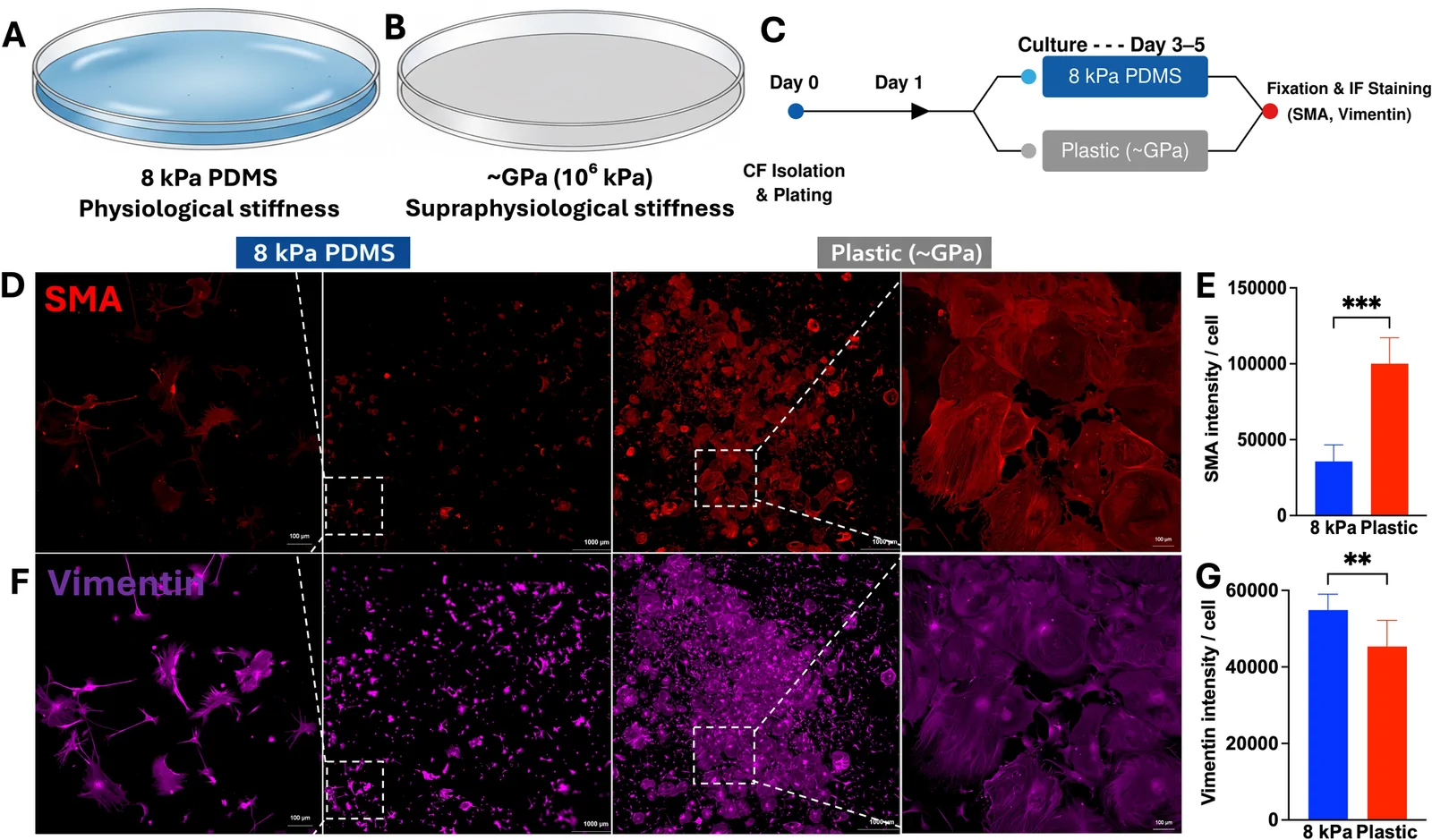
Matrix stiffness alters the baseline cytoskeletal state of cardiac fibroblasts. **(A, B)** Schematic comparison of an 8 kPa PDMS substrate and conventional tissue-culture plastic (∼GPa). **(C)** Experimental timeline. Cardiac fibroblasts (CFs) were isolated from neonatal *Gata4^fl/fl^* mice and cultured on either substrate for 3–5 days before fixation and immunofluorescence staining. **(D, E)** Representative *α*-SMA images and quantification of fluorescence intensity per cell. **(F, G)** Representative Vimentin images and quantification. The outer images in panels D and F are enlarged views of the boxed regions in the corresponding whole-dish images. Images are stitched composites acquired at 10× magnification; fluorescence intensity per cell was quantified using ImageJ. Data are mean ± SEM; *n* = 5 biological preparations per group. Unpaired Student's t-tests; **p < 0.01 and ***p < 0.001. Scale bar, 1000 µm. Both sexes were included.

### RNA extraction and quantitative RT-PCR

Total RNA was extracted using TRIzol Reagent (Thermo Fisher, cat# 15-596-018). Complementary DNA was synthesized using the High-Capacity cDNA Reverse Transcription Kit (Applied Biosystems, cat# 4368814). Quantitative PCR was performed using SsoAdvanced Universal SYBR Green Supermix (Bio-Rad, cat# 1725275) on a Quantagene q225 Real-Time PCR System. Gene expression was normalized to Gapdh using the 2^−*ΔΔ*Ct^ method. Primer sequences are provided in Table 1.

**Table 1:** qRT-PCR primer sequences.

Gene	Forward Primer (5′→3′)	Reverse Primer (5′→3′)
*Gata4*	GCCGAGGGAGCCGCCTACAC	TGGGGTGTCTTCCAGGGTTGG
*Col1a1*	CCTCAGGGTATTGCTGGACAAC	CAGAAGGACCTTGTTTGCCAGG
*Col1a2*	TTCTGTGGGTCCTGCTGGGAAA	TTGTCACCTCGGATGCCTTGAG
*Col3a1*	GACCAAAAGGTGATGCTGGACAG	CAAGACCTCGTGCTCCAGTTAG
*Gapdh*	CATCACTGCCACCCAGAAGACTG	ATGCCAGTGAGCTTCCCGTTCAG

### Live-cell migration assay

Migratory behavior was assessed by time-lapse phase-contrast microscopy. Cells were seeded at low density (2 × 103 cells/dish) on 8 kPa PDMS dishes. Images were acquired every 10 min over 12–16 h using a Nikon Eclipse Ti2 live-cell imaging system. Cell trajectories were analyzed using AI-assisted binary segmentation (NIS-Elements AR). Per-cell outputs included instantaneous speed, path speed, line speed, mean cell area, segment length, and directional persistence. In NIS-Elements, Speed is calculated for each trajectory segment as the segment length divided by the elapsed time since the preceding frame. In this manuscript, instantaneous speed refers to the per-cell mean of these stepwise Speed values; therefore, Speed and instantaneous speed are not separate measurements. Path speed was calculated as cumulative path length divided by total tracking duration, whereas line speed was calculated as the straight-line displacement from the initial to the final position divided by total tracking duration. Directional persistence was calculated as line length divided by cumulative path length. Values approaching 1 indicate persistent directional movement, whereas values approaching 0 indicate a less direct trajectory with little net displacement. Segment length was the distance between two consecutive centroid positions. Path distance and net displacement were standardized to a common 1-h interval using the corresponding path speed and line speed, respectively. Normalized path distance was calculated as path speed  ×  3,600 s, and normalized net displacement was calculated as line speed  ×  3,600 s. Only cells tracked for at least 36 consecutive time points (≥6 h) without division were included. Raw cumulative path length and line length were not used for biological interpretation because cells were retained after a minimum rather than identical tracking interval.

### RNA-seq data analysis

Adult mouse cardiac fibroblast RNA-seq data were obtained from GSE155358 (29). Only the three control and three single-*Gata4*-knockout samples were included. Author-provided gene-level fold changes were reoriented as *Gata4* knockout versus control. Twenty-four prespecified GO Biological Process 2023 gene sets were grouped into four migration domains: motility, actin remodeling, polarity/protrusion, and adhesion coupling. Preranked gene-set enrichment analysis was performed using the *Gata4*-knockout-versus-control fold-change ranking, 4,000 random gene-set permutations, and Benjamini–Hochberg correction across the 24 tested terms (31, 32). Normalized enrichment scores (NES) and false-discovery rates (FDR) are reported. Sample-level rank scores were calculated by ranking genes within each sample, averaging ranks for genes in each set, centering the result, and standardizing scores within each domain for visualization. Selected actin assembly, turnover, contractility, adhesion-coupling, and polarity genes were examined descriptively using *Gata4*-knockout-versus-control log2 fold changes; individual genes were not assigned significance from fold change alone.

### Migration effect-size integration

For the cross-source migration experiment, each metric was extracted directly from the Prism grouped-data tables, retaining the available number of cell observations for each metric. To compare migration parameters expressed in different units, standardized mean differences between *Gata4* KO and Control cells were calculated separately for each fibroblast source and metric using Hedges’ g, based on the pooled within-group standard deviation with a small-sample correction. The contrast was defined as *Gata4* KO minus Control; therefore, negative values indicated a reduction and positive values indicated an increase following *Gata4* deletion. Ninety-five percent confidence intervals were estimated by resampling Control and *Gata4* KO cells within each fibroblast source and metric 10,000 times using a fixed random seed. Effect sizes were used descriptively to summarize the direction and relative magnitude of changes across migration parameters and did not replace the primary groupwise statistical comparisons. Rate, distance, directional-persistence, and cell-geometry measurements were displayed together, with cumulative path and line lengths interpreted alongside time-normalized migration parameters (33).

### Statistical analysis

qRT-PCR expression was calculated using the 2^−*ΔΔ*Ct^ method with *Gapdh* as the reference gene. Two-group comparisons used unpaired Student's *t*-tests, whereas experiments involving multiple fibroblast sources and treatments used two-way ANOVA with Sidak's multiple-comparisons test. For migration analyses, each dot represents one tracked cell, with biological replicate numbers provided in the figure legends. The relationship between mean cell area and instantaneous speed was analyzed separately in Control and Gata4-knockout CFs using Spearman rank correlation. Hedges’ *g* and 10,000 bootstrap resamples were used to descriptively compare effects across migration parameters. RNA-seq FDR values were adjusted across the 24 tested gene sets using the Benjamini–Hochberg method. *P* or FDR < 0.05 was considered significant. Experimental analyses were performed using GraphPad Prism version 10.

## Results

### 8 kPa PDMS substrates limit stiffness-induced cytoskeletal activation in cardiac fibroblasts

To establish a physiologically relevant culture system, we compared CF phenotype on 8 kPa PDMS substrates versus conventional tissue culture plastic (∼GPa; Figures 1A,B). Reported myocardial stiffness values depend on the measurement method. The Young's modulus of diastolic adult human myocardium has been reported to be approximately 8–15 kPa, while cardiac magnetic resonance elastography measured values of 7.2–11.8 kPa, with a median of 8.2 kPa, in healthy volunteers (14, 15), whereas standard tissue culture plastic is several orders of magnitude stiffer, a discrepancy known to drive spontaneous MF activation *in vitro*. CFs were isolated from neonatal *Gata4^fl/fl^* mice, cultured on either substrate for 3–5 days prior to fixation and immunofluorescence staining for markers of fibroblast activation and identity (Figure 1C). CFs cultured on plastic exhibited significantly higher *α*-SMA expression, a canonical marker of MF activation and stress fiber assembly, compared with CFs maintained on 8 kPa PDMS (*p* < 0.001, Figures 1D,E). Conversely, Vimentin, a fibroblast intermediate-filament protein, was significantly elevated in CFs on 8 kPa substrates relative to plastic (*p* < 0.01, Figures 1F,G). Increased *α*-SMA intensity, filamentous stress-fiber-like organization, and increased cell spreading were interpreted as features of a more activated, myofibroblast-like cytoskeletal state. Vimentin was used to visualize the intermediate-filament cytoskeleton and was not used alone as an activation marker. These data indicate that 8 kPa PDMS substrates limit spontaneous CF activation *in vitro*.

### 8 kPa substrate stiffness preserves cardiac fibroblast migratory capacity

To determine whether substrate stiffness influences CF migratory behavior, we compared CFs cultured on 8 kPa PDMS substrates with those on standard tissue culture plastic. Cell trajectories were tracked by live-cell time-lapse imaging. Instantaneous speed represents the per-cell mean of the stepwise Speed values between consecutive frames. Path speed is the cumulative path length divided by total tracking duration, whereas line speed is the straight-line displacement from the initial to the final position divided by total tracking duration. Directional persistence is the ratio of line length to cumulative path length, with values approaching 1 indicating persistent directional movement and values approaching 0 indicating a less direct trajectory. CFs on 8 kPa substrates exhibited significantly higher instantaneous speed (Figure 2A, *p* < 0.0001), path speed (Figure 2B, *p* < 0.0001), line speed (Figure 2D, *p* < 0.0001), and segment length (Figure 2G, *p* < 0.0001) compared to cells on plastic. Directional persistence was also greater on 8 kPa substrates (Figure 2H, *p* < 0.001), and net displacement (line length) was significantly increased (Figure 2C, *p* < 0.01). Total path length did not differ significantly between conditions (Figure 2E, ns). In contrast, CFs on plastic displayed markedly larger cell area (Figure 2F, *p* < 0.0001), consistent with the MF-like morphology induced by supraphysiological stiffness. Together, these data indicate that plastic-activated CFs adopt a spread, MF-like phenotype and exhibit reduced migration speed and directional persistence relative to CFs maintained on 8-kPa PDMS substrates.

**Figure 2:**
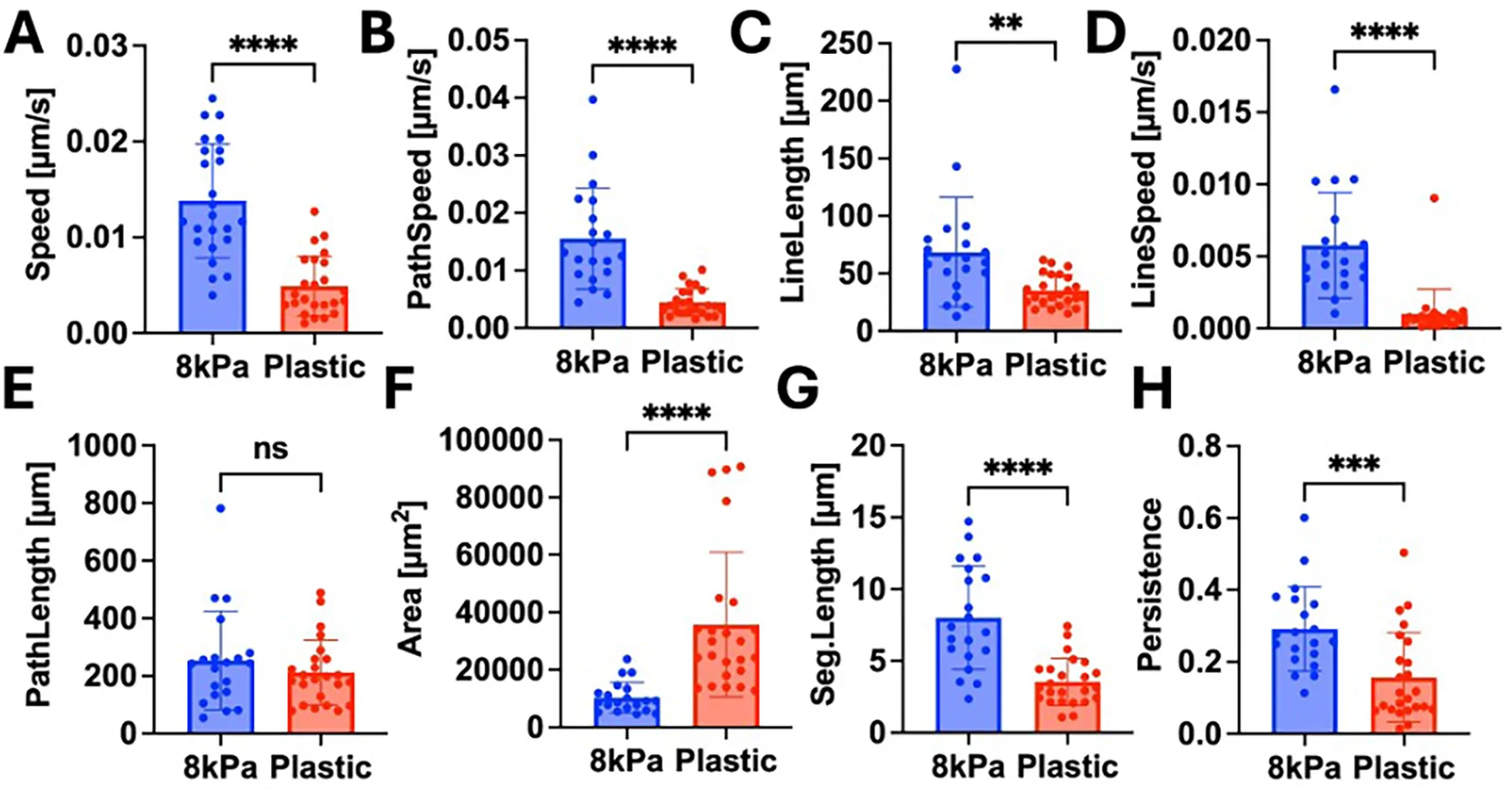
8-kPa PDMS substrates preserve cardiac fibroblast migratory capacity. Cardiac fibroblasts isolated from neonatal *Gata4^fl/fl^* mice were cultured on 8 kPa PDMS or conventional tissue-culture plastic and imaged for 12–16 h. **(A)** Instantaneous speed, **(B)** path speed, **(C)** net displacement (line length), **(D)** line speed, **(E)** cumulative path length, **(F)** cell area, **(G)** segment length, and **(H)** directional persistence. Bars are mean ± SEM; dots are tracked cells from five biological preparations per condition. Unpaired Student's t-tests; **p < 0.01, ***p < 0.001, ****p < 0.0001; ns, not significant. Both sexes were included.

### GATA4 is differentially expressed across fibroblast subtypes and efficiently deleted by Ad-Cre

We next characterized baseline GATA4 expression across the three fibroblast populations and examined the transcriptional consequences of its deletion. qRT-PCR analysis revealed a striking tissue-specific gradient: CFs expressed markedly higher levels of *Gata4* mRNA than LFs (*p* < 0.001, Figure 3A), which in turn expressed significantly higher levels than TTFs (*p* < 0.01, Figure 3A), and both were significantly higher than TTFs relative to CFs (*p* < 0.0001, Figure 3A). This gradient demonstrates tissue-source-dependent differences in baseline *Gata4* expression and suggests that the contribution of GATA4 may differ among fibroblast populations. To achieve cell-autonomous GATA4 deletion, fibroblasts were transduced with adenoviral Cre recombinase (Ad-Cre-GFP) or control vector (Ad-GFP). Adenoviral Cre-mediated deletion reduced *Gata4* mRNA expression by >90% in all three fibroblast sources (CF: *p* < 0.0001; LF: *p* < 0.0001; TTF: *p* < 0.001; Figure 3B), confirming efficient deletion across fibroblast subtypes regardless of baseline expression level.

**Figure 3:**
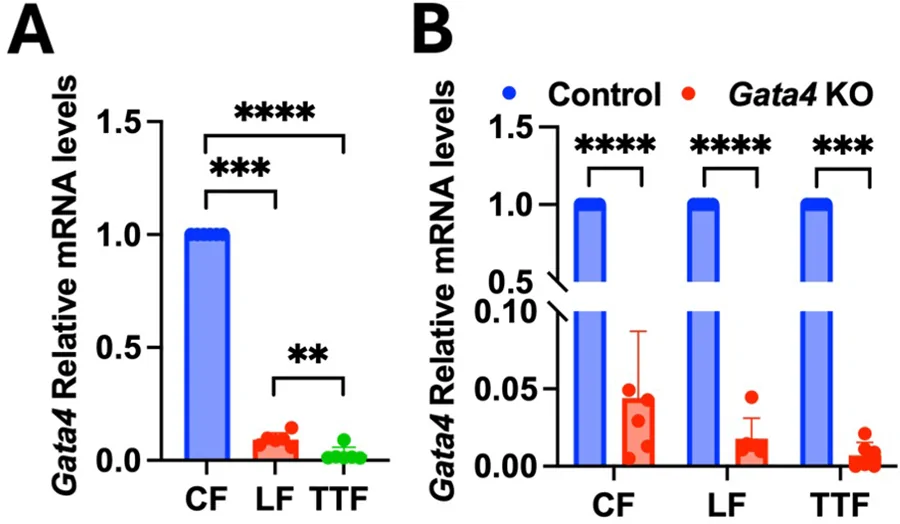
GATA4 abundance differs among fibroblast sources and is efficiently reduced by Ad-Cre. CFs, LFs, and TTFs isolated from neonatal *Gata4^fl/fl^* mice were cultured on 8 kPa PDMS. **(A)** Baseline *Gata4* mRNA expression across fibroblast sources, normalized to CFs. **(B)** *Gata4* mRNA expression after Ad-GFP (Control) or Ad-Cre-GFP (*Gata4* KO) transduction. Gene expression was measured by qRT-PCR and normalized to *Gapdh*. Data are mean ± SEM; *n* = 5 biological preparations per group. Unpaired Student's t-tests; **p < 0.01, ***p < 0.001, ****p < 0.0001. Both sexes were included.

### *Gata4* deletion increases collagen gene expression across fibroblast subtypes

Fibroblast migration during tissue repair occurs alongside remodeling of the surrounding extracellular matrix. To determine whether the effects of GATA4 loss extended beyond cell behavior to matrix-related gene expression, we examined selected structural collagen genes following GATA4 deletion in CFs, LFs, and TTFs. qRT-PCR showed that *Gata4* deletion significantly increased *Col1a1, Col1a2*, and *Col3a1* mRNA levels across CFs, LFs, and TTFs (Figures 4A–C). Despite their distinct tissue origins and different baseline levels of GATA4, all three fibroblast populations showed a consistent increase in the selected collagen transcripts following GATA4 deletion. These findings suggest that GATA4 contributes to the regulation of matrix-related gene expression across fibroblast populations and provide ECM-remodeling context for the cell migration analyses described below. However, changes in these selected transcripts alone do not establish a global pro-fibrotic state or a causal relationship between collagen expression and migration.

**Figure 4:**
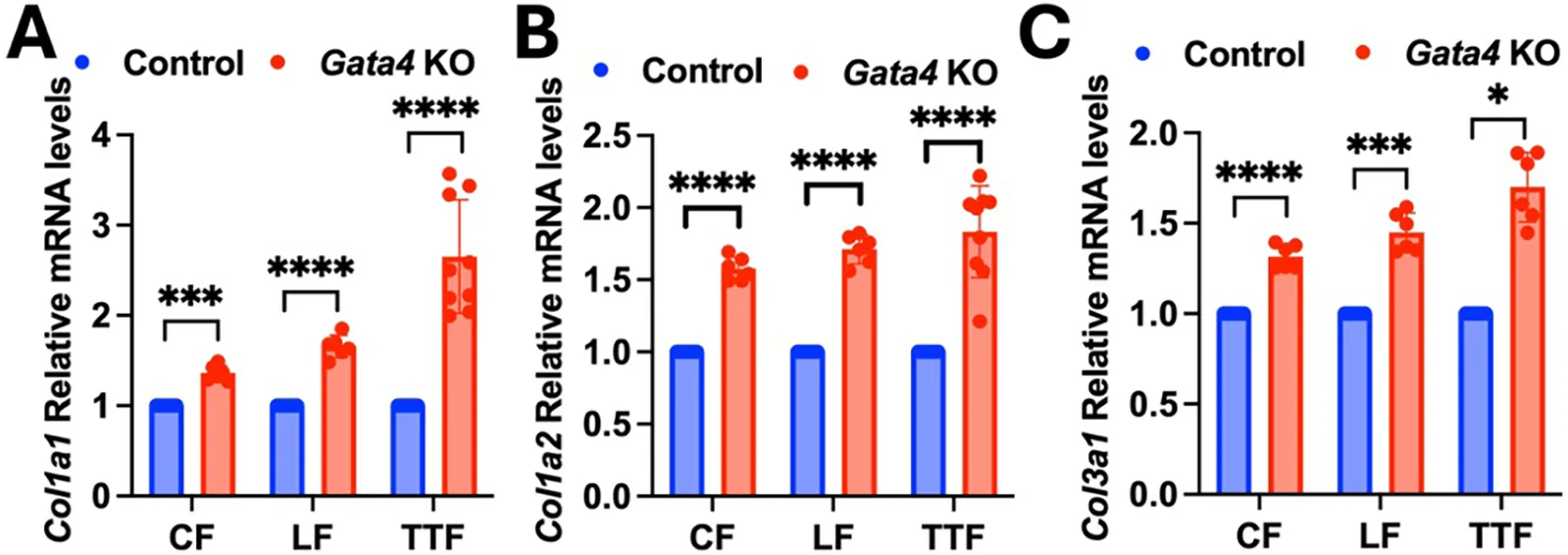
*Gata4* deletion increases selected collagen mRNA levels across fibroblast sources. CFs, LFs, and TTFs were cultured on 8 kPa PDMS and transduced with Ad-GFP (Control) or Ad-Cre-GFP (*Gata4* KO). qRT-PCR measurement of **(A)** *Col1a1*, **(B)** *Col1a2*, and **(C)** *Col3a1* mRNA, normalized to *Gapdh*. Data are mean ± SEM; *n* = 5 biological preparations per group. Unpaired Student's t-tests; *p < 0.05, ***p < 0.001, ****p < 0.0001. Both sexes were included.

### GATA4 deletion alters fibroblast migration in a fibroblast-source-dependent manner

To determine whether GATA4 regulates fibroblast migration in a fibroblast-source-dependent manner, we performed live-cell tracking of CFs, LFs, and TTFs following Ad-Cre or Ad-GFP transduction on 8 kPa PDMS substrates (Figure 5). To permit direct comparison of distance-based outcomes across trajectories, path distance and net displacement were standardized to a common 1-h interval using the corresponding path speed and line speed, respectively. GATA4 deletion produced distinct migratory phenotypes across the three fibroblast subtypes.

**Figure 5:**
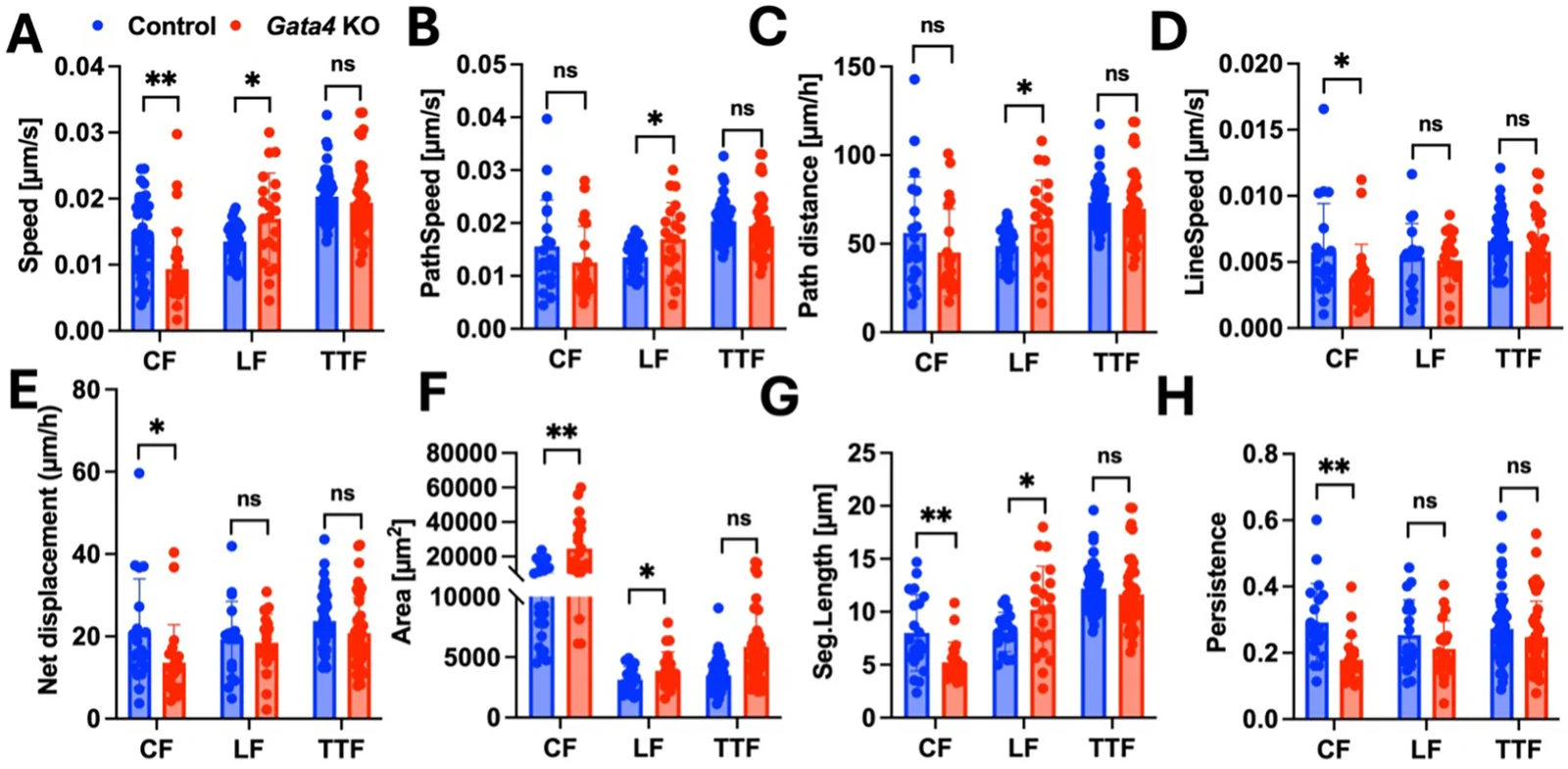
*Gata4* deletion remodels fibroblast migration in a fibroblast-source-dependent manner. CFs, LFs, and TTFs isolated from neonatal *Gata4^fl/fl^* mice were cultured on 8 kPa PDMS and transduced with Ad-Cre-GFP (*Gata4* KO) or Ad-GFP (Control). **(A)** Instantaneous speed, **(B)** path speed, **(C)** path distance, **(D)** line speed, **(E)** net displacement, **(F)** cell area, **(G)** segment length, and **(H)** directional persistence. Points are individual tracked cells; black bars show the interquartile range and white ticks the median. Metric-specific n values are displayed beneath each cell source as Control/KO. Statistical significance from two-way ANOVA with Sidak's multiple-comparisons test is indicated by *p < 0.05 and **p < 0.01; ns, not significant. Standardized effects and bootstrap confidence intervals are summarized in Figure 6. Both sexes were included.

In CFs, *Gata4* KO significantly reduced instantaneous speed (Figure 5A, *p* < 0.01), line speed (Figure 5D, *p* < 0.05), normalized net displacement (Figure 5E, *p* < 0.05), segment length (Figure 5G, *p* < 0.01), and directional persistence (Figure 5H, *p* < 0.01), indicating slower and less persistent movement. Path speed and normalized path distance were not significantly altered. Instantaneous speed summarizes local movement between consecutive frames, whereas path speed represents cumulative movement over the complete trajectory. Line speed measures net directional advance and equals path speed multiplied by directional persistence. Therefore, reduced directional persistence can decrease line speed even when path speed is not significantly altered. These results indicate reduced local movement and less efficient conversion of cumulative movement into net displacement, rather than a uniform reduction in all measures of movement. Cell area was significantly increased following *Gata4* deletion (Figure 5F, *P* < 0.01). Track-matched analysis showed that cell area was positively correlated with instantaneous speed in Control CFs (*ρ* = 0.45, *P* = 0.047, *n* = 20) and *Gata4*-knockout CFs (*ρ* = 0.65, *P* = 0.0015, *n* = 21; Figure 6E). Thus, GATA4-deficient CFs exhibited slower movement, reduced directional advance, and lower migratory persistence while adopting a more spread morphology.

**Figure 6:**
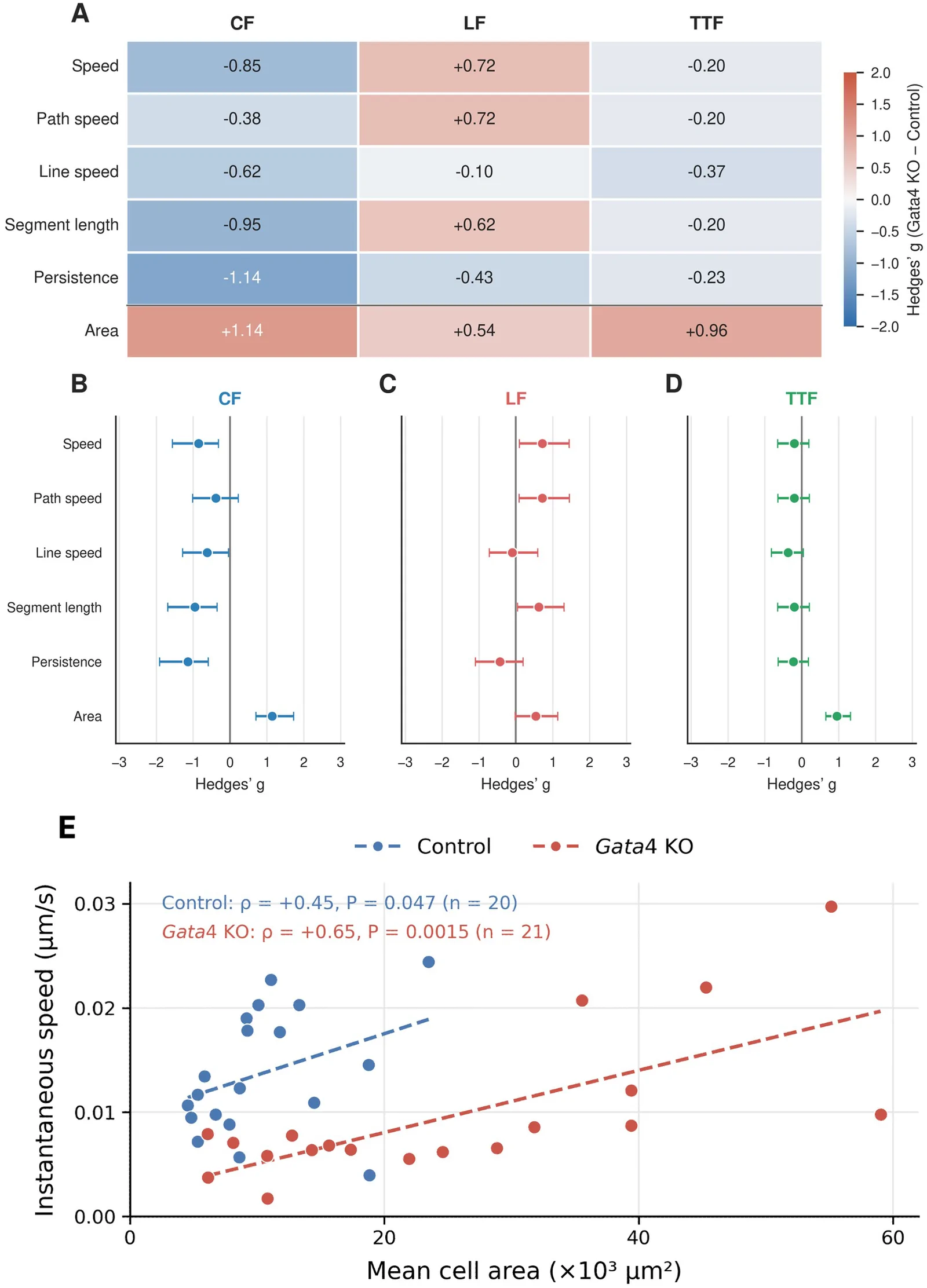
Integrated effect-size analysis highlights fibroblast-source-dependent migration responses. **(A)** Heatmap of Hedges’ g calculated as *Gata4* KO minus control for duration-comparable migration measurements and cell area. Negative values indicate lower measurements after *Gata4* deletion; positive values indicate higher measurements. **(B–D)** Forest plots show Hedges’ g and 95% bootstrap confidence intervals for CFs, LFs, and TTFs. The vertical line at g = 0 indicates no difference. Cumulative path and line lengths were excluded from the integrated analysis because of their sensitivity to effective tracking duration. Effect sizes are descriptive summaries of cell-level distributions. **(E)** Track-matched relationship between mean cell area and instantaneous speed in Control and *Gata4*-knockout CFs. Each point represents one tracked cell. Spearman rank correlations were calculated separately for Control (*n* = 20) and *Gata4*-knockout CFs (*n* = 21).

In LFs, GATA4 deletion produced a more modest phenotype, with significant increases in instantaneous speed (Figure 5A, *p* < 0.05), path speed (Figure 5B, *p* < 0.05), normalized path distance (Figure 5C, *p* < 0.05), segment length (Figure 5G, *p* < 0.05), and cell area (Figure 5F, *p* < 0.05). Line speed, normalized net displacement, and directional persistence were not significantly altered, indicating that GATA4 loss primarily increased local migration rate and cell spreading in LFs without improving directional efficiency.

In TTFs, *Gata4* deletion did not significantly alter instantaneous speed, path speed, normalized path distance, line speed, normalized net displacement, segment length, or directional persistence. Thus, TTF migration was comparatively insensitive to *Gata4* loss under these experimental conditions.

Collectively, these results reveal that GATA4 deletion produces distinct migratory responses depending on fibroblast source, with the most coordinated reduction in migration rate, directional advance, and persistence observed in CFs.

### Integrated effect-size analysis highlights fibroblast-source-dependent migration responses

Because the migration parameters were measured in different units, we calculated Hedges’ g to compare the direction and magnitude of the GATA4-dependent effects across metrics (Figure 6). Hedges’ g was calculated as *Gata4* KO minus Control; therefore, negative values indicate lower measurements after *Gata4* deletion, whereas positive values indicate higher measurements. The effect-size heatmap summarizes the response patterns across the three fibroblast sources (Figure 6A), while the corresponding forest plots show the effect-size estimates and 95% bootstrap confidence intervals for CFs, LFs, and TTFs, respectively (Figures 6B–D). In the forest plots, the vertical line at Hedges’ g = 0 represents no difference between groups. Values to the left indicate lower measurements in *Gata4* KO cells, whereas values to the right indicate higher measurements. The integrated analysis focused on duration-comparable migration parameters, including instantaneous speed, path speed, line speed, segment length, and directional persistence, together with cell area as a morphological measurement. Cumulative path and line lengths were not included because these measurements are sensitive to effective trajectory duration. In CFs, *Gata4* deletion produced relatively large reductions in instantaneous speed (g = −0.85), line speed (g = −0.62), segment length (g = −0.95), and directional persistence (g = −1.14), together with a marked increase in cell area (g =  + 1.14; Figure 6B). This pattern indicates that GATA4-deficient CFs moved more slowly, formed shorter migration segments, and migrated with lower directional persistence while adopting a more spread morphology. LFs exhibited a different response. *Gata4* deletion increased instantaneous speed and path speed (both g =  + 0.72) and segment length (g =  + 0.62; Figure 6C). However, the effect on directional persistence was smaller, and its bootstrap confidence interval included zero, indicating no clear improvement in directional migration. In TTFs, the effects on instantaneous speed (g = −0.20), path speed (g = −0.20), line speed (g = −0.37), segment length (g = −0.20), and directional persistence (g = −0.23) were small (Figure 6D). In contrast, the effect-size estimate for cell area was positive (g =  + 0.96), suggesting that the morphological response was greater than the migration response. Together, the effect-size analysis demonstrates that *Gata4* deletion produces fibroblast-source-dependent responses. CFs showed the most coordinated reduction in migration rate and directional persistence, LFs primarily exhibited increased local movement without a clear improvement in directionality, and TTFs showed little change in duration-normalized migration parameters.

### *Gata4* loss induces non-uniform remodeling of migration-related transcriptional programs in adult cardiac fibroblasts

To determine whether the altered CF migration phenotype was accompanied by coordinated transcriptional remodeling, we analyzed publicly available adult cardiac fibroblast RNA-seq data from GSE155358, restricting the comparison to control and single-*Gata4*-knockout samples (*n* = 3 per group). A focused panel of 24 migration-related Gene Ontology Biological Process gene sets was examined across four functional domains: motility/migration, actin remodeling, polarity/protrusion, and adhesion coupling. Positive normalized enrichment scores (NES) indicate enrichment toward genes increased after *Gata4* loss. Positive regulation of cell motility (NES = 1.89, FDR = 0.0029), positive regulation of cell migration (NES = 1.88, FDR = 0.0029), and regulation of cell migration (NES = 1.70, FDR = 0.0029) were significantly enriched after *Gata4* deletion (Figure 7A). Actomyosin structure organization (NES = 1.82, FDR = 0.0049), actin cytoskeleton reorganization (NES = 1.66, FDR = 0.021), and actin filament organization (NES = 1.55, FDR = 0.021) were also enriched. In contrast, none of the polarity/protrusion or adhesion-coupling gene sets reached statistical significance at FDR < 0.05. Domain-level integration therefore showed stronger and more coordinated transcriptional changes in the motility/migration and actin-remodeling domains than in the polarity/protrusion and adhesion-coupling domains (Figure 7B). Consistent with this result, rank-based domain scores showed higher expression scores for motility-associated and actin-remodeling gene sets in the *Gata4*-knockout samples, whereas polarity/protrusion scores were modestly lower and adhesion-coupling scores remained comparatively similar between groups (Figure 7C). These sample-level scores are presented descriptively because of the limited number of biological replicates. Collectively, these results indicate that *Gata4* loss induces non-uniform remodeling of migration-related transcriptional programs rather than a coordinated pro-migratory state. Actin- and motility-associated genes were enriched without corresponding activation of the polarity, protrusion, and adhesion-turnover processes required for efficient directional migration, consistent with the experimentally observed reduction in CF migration speed and directional persistence.

**Figure 7:**
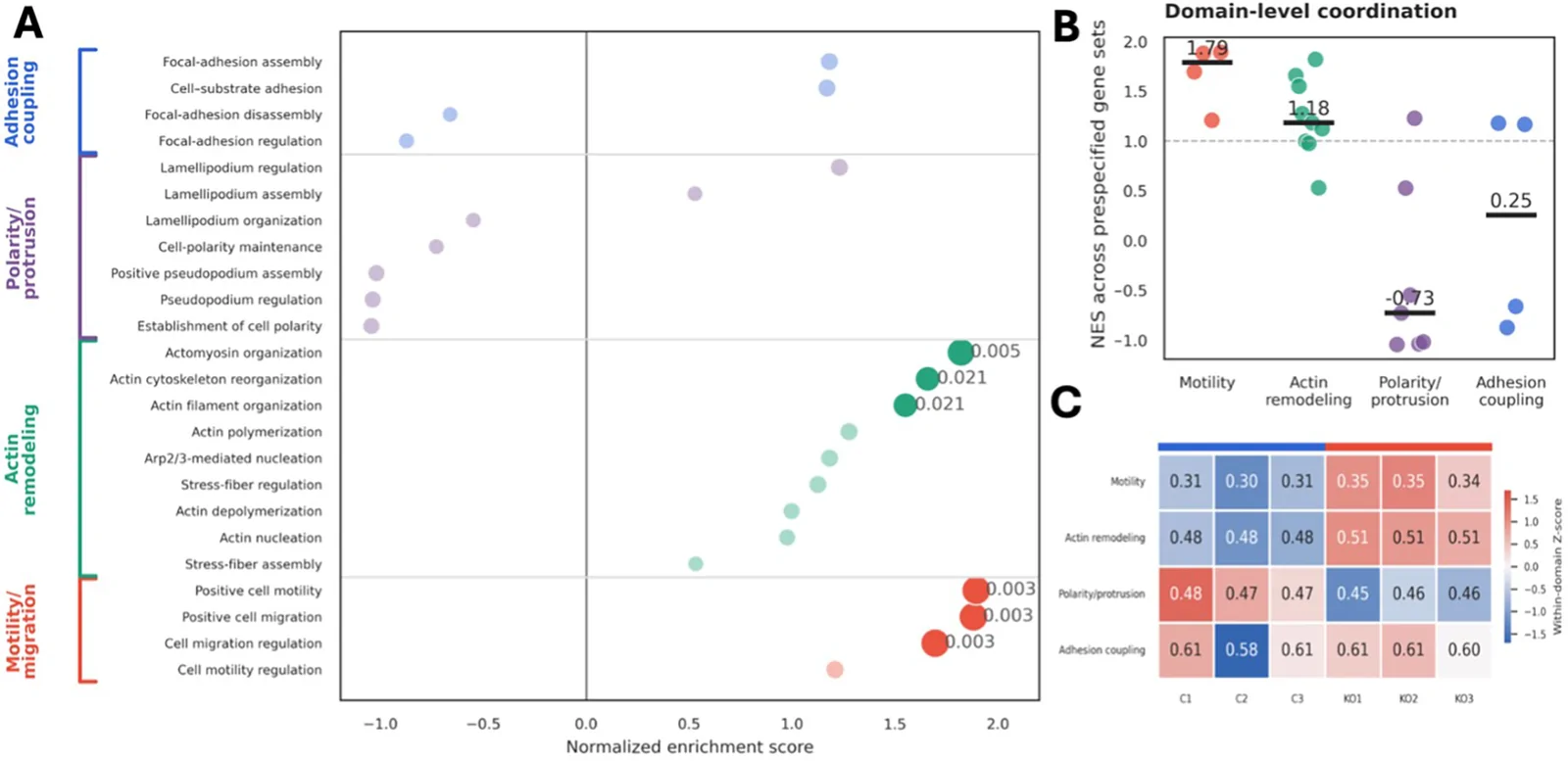
*Gata4* loss produces non-uniform remodeling of migration-related transcriptional programs. Publicly available adult cardiac fibroblast RNA-Seq data from GSE155358 were analyzed using control and single-*Gata4*-knockout samples (*n* = 3 per group). **(A)** NES values for 24 focused GO Biological Process gene sets grouped into motility/migration, actin remodeling, polarity/protrusion, and adhesion coupling. FDR values are labeled for terms with FDR < 0.05. **(B)** Distribution of NES values within each functional domain; black lines and numbers indicate domain medians. **(C)** Rank-based domain scores across individual control (C1–C3) and knockout (KO1–KO3) samples. Cell colors show within-domain standardized values; numbers show the underlying rank-based scores. Absence of significant enrichment is not interpreted as pathway suppression.

### *Gata4* loss differentially remodels actin cytoskeletal submodules

Gene-level analysis further demonstrated that the transcriptional response involved multiple components of the actin and migration machinery (Figure 8). Core actin genes, including *Acta2, Actg1,* and *Actb*, showed positive log2 fold changes after *Gata4* deletion (Figure 8A). Genes associated with actin polymerization and branching, including *Enah, Vasp, Actr3,* and *Pfn1*, also showed positive changes. Actin-turnover genes changed in different directions: *Dstn* and *Cfl1* increased, whereas *Gsn* decreased. Contractile and stress-fiber-associated genes, including *Tagln*, *Tpm1, Tpm2*, and *Mylk*, were increased, together with positive changes in several individual adhesion-associated genes. In contrast, the polarity-related small *GTPases Cdc42, Rac1,* and *Rhoa* showed only small negative changes. The individual-sample heatmap showed that the overall expression patterns of these actin submodules were generally consistent across samples within each group rather than being confined to a single sample (Figure 8B). Individual-gene fold changes are interpreted descriptively; however, their combined pattern indicates extensive remodeling of actin assembly, turnover, and contractile machinery, with changes in selected adhesion components but without a coordinated polarity response. This pattern is consistent with the increased cell area and reduced migration speed and directional persistence observed in *Gata4*-deficient CFs.

**Figure 8:**
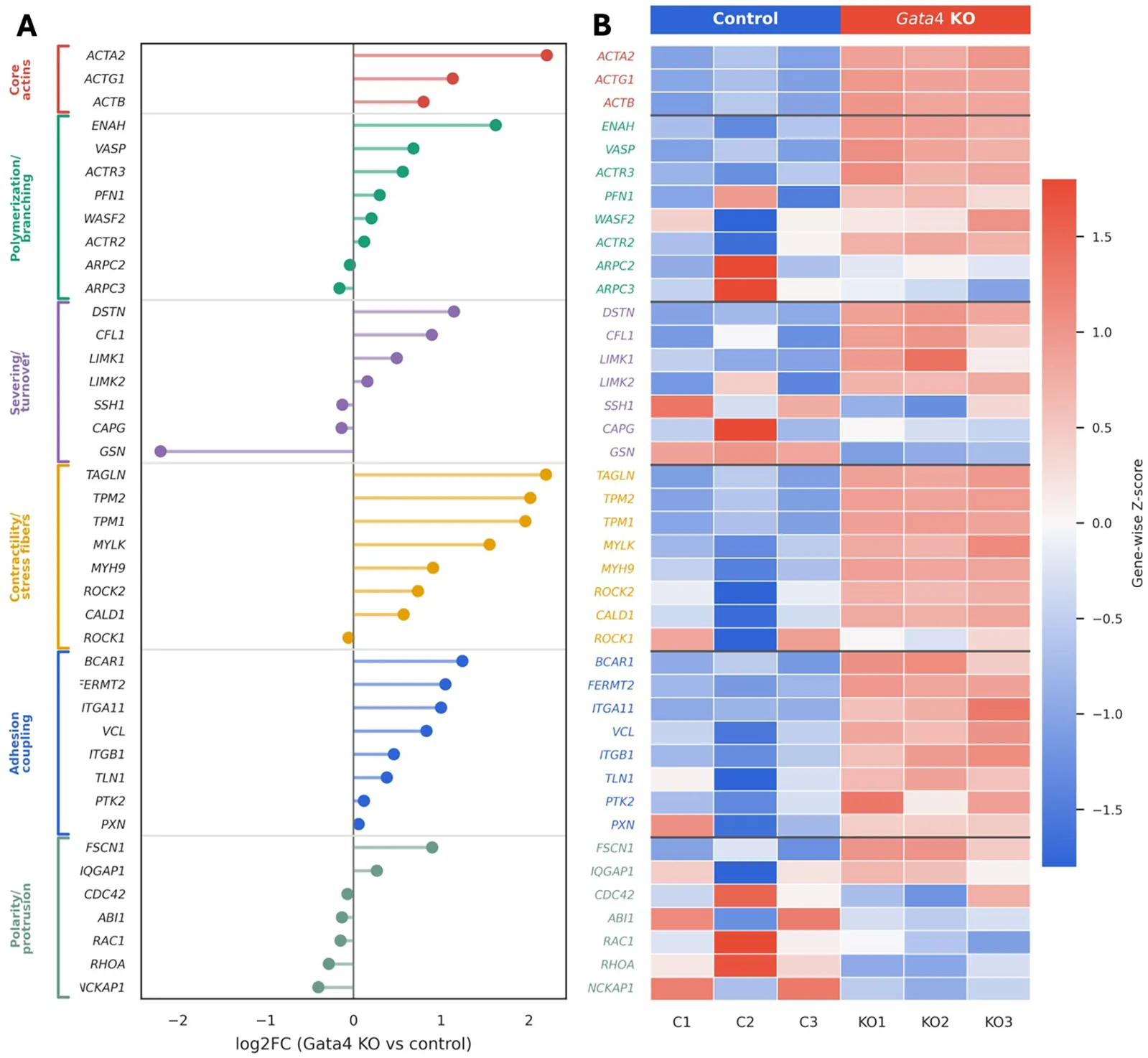
*Gata4* loss differentially remodels actin cytoskeletal submodules. **(A)** *Gata4*-knockout-versus-control log2 fold changes for selected genes grouped into core actins, polymerization/branching, severing/turnover, contractility/stress fibers, adhesion coupling, and polarity/protrusion. **(B)** Gene-wise z-score heatmap across the three control (C1–C3) and three *Gata4*-knockout (KO1–KO3) samples. Individual-gene fold changes are displayed descriptively and are not assigned statistical significance from fold change alone.

## Discussion

The present study identifies GATA4 as a fibroblast-source-dependent regulator of fibroblast migration under physiologically relevant mechanical conditions. Using 8 kPa PDMS substrates, we found that *Gata4* expression was substantially higher in CFs than in LFs or TTFs. *Gata4* deletion increased selected collagen transcripts across all three fibroblast populations but produced distinct migratory responses depending on tissue of origin. The most coordinated reductions in migration speed and directional persistence occurred in CFs. Analysis of an independent adult CF RNA-seq dataset further associated *Gata4* loss with actomyosin- and actin-remodeling-biased transcriptional changes without coordinated activation of polarity and adhesion-turnover programs. Together, these findings suggest that GATA4 helps coordinate cytoskeletal remodeling with efficient and persistent fibroblast migration, with a particularly important role in the cardiac lineage. Recent studies have highlighted KLF1/Wnt/*β*-catenin signaling and the myocardial microenvironment as broader regulators of cardiac repair (34, 35). In cardiomyocytes, KLF1 overexpression increased chromatin accessibility at regions enriched for GATA4 motifs (35). However, KLF1 was reported to be expressed predominantly in cardiomyocytes, and these data do not establish KLF1 as a direct upstream regulator of GATA4 in fibroblasts (35). Whether signals from this broader cardiac transcriptional network regulate GATA4 expression or activity in CFs remains to be determined.

The use of 8-kPa PDMS substrates is a critical methodological feature of this study. As demonstrated in Figure 1, standard tissue culture plastic induced a more spread, *α*-SMA-rich cytoskeletal phenotype, whereas Vimentin staining demonstrated additional substrate-dependent remodeling of the intermediate-filament network, confounding the interpretation of experiments aimed at understanding CF-intrinsic regulatory mechanisms. By culturing cells on 8 kPa PDMS, we were able to isolate the effects of GATA4 deletion from the background of substrate-induced activation. This is further supported by the migration data in Figure 2, which reveal that plastic-cultured CFs, despite their activated morphology and markedly enlarged cell area, exhibit significantly reduced instantaneous speed, path speed, line speed, segment length, and directional persistence compared with CFs on 8 kPa substrates. This paradox—whereby MF activation is associated with reduced rather than enhanced motility—is consistent with the well-established role of mature focal adhesions and prominent stress fiber networks in restraining random cell motility (10, 20). Activated MFs are optimized for contractile force generation rather than translocation, and the supraphysiological stiffness of plastic may further reinforce this phenotype through mechanotransductive feedback (16, 20). Together, Figures 1, 2 validate our experimental platform and establish that 8 kPa PDMS substrates preserve a less activated cytoskeletal state and greater intrinsic migratory capacity of CFs *in vitro*.

*Gata4* deletion increased *Col1a1, Col1a2*, and *Col3a1* mRNA levels expression across CFs, LFs, and TTFs (Figure 4). However, the migratory effects of *Gata4* deletion differed substantially among the three cell sources despite their shared collagen response. Therefore, changes in migration and ECM-related gene expression should not be considered equivalent consequences of *Gata4* loss. Moreover, changes in these selected collagen transcripts alone do not establish a global pro-fibrotic state or demonstrate that altered collagen expression directly causes the migration phenotype. The migration analyses revealed clear fibroblast-source-dependent responses to *Gata4* deletion (Figures 5, 6). In CFs, *Gata4* loss reduced instantaneous speed, line speed, segment length, and directional persistence while increasing cell area. Path speed and normalized 1-h path distance were not significantly altered. The integrated effect-size analysis confirmed that CFs showed the most coordinated reduction in migration rate and directionality. LFs exhibited increases in instantaneous speed, path speed, normalized 1-h path distance, and segment length, but no clear improvement in line speed, normalized net displacement, or directional persistence. TTFs showed little change across the time-normalized migration parameters. Standardizing the distance measurements to a common 1-h interval removed the apparent inconsistency between speed and cumulative distance in the original analysis. Overall, increased cell area co-occurred with reduced speed and persistence in *Gata4*-deficient CFs at the group level. However, track-matched analysis showed a positive relationship between cell area and instantaneous speed within both Control and *Gata4*-deficient CFs. Thus, increased cell area and altered migration are interpreted as parallel consequences of *Gata4* loss rather than evidence that greater cell spreading causes the migration defect. The adult CF RNA-seq analysis provides a transcriptional framework for interpreting the CF migration phenotype. Gene sets annotated as positive regulation of motility and migration were enriched after *Gata4* loss, but this enrichment should not be interpreted as evidence of increased functional migration. These broad annotations include genes involved in cytoskeletal remodeling and contractility. Consistent with this interpretation, actomyosin organization, actin cytoskeleton reorganization, and actin filament organization were enriched, whereas polarity/protrusion and adhesion-coupling programs were not comparably enriched (Figure 7). Gene-level analysis further showed positive changes in core actins and polymerization-associated genes, together with increased expression of several contractile and stress-fiber-associated genes (Figure 8). In contrast, the polarity-related small GTPases Cdc42, Rac1, and Rhoa did not show a corresponding positive response. Together, the RNA-seq and live-cell migration data support a model in which GATA4 coordinates actomyosin remodeling with cell polarity and adhesion turnover to sustain efficient and persistent CF migration. *Gata4* loss did not globally suppress migration-related transcription; instead, it shifted the transcriptional program toward actomyosin assembly and contractility without parallel engagement of polarity/protrusion and adhesion-turnover modules. This imbalance provides a plausible explanation for the enlarged, spread morphology and reduced migration speed and directional persistence of GATA4-deficient CFs. Thus, the principal consequence of *Gata4* loss may not be a simple reduction in the migration machinery, but rather a loss of coordination between force-generating cytoskeletal remodeling and directional cell translocation. The direct GATA4 targets and causal intermediates responsible for this coupling remain to be established.

It is important to note that the migration assay employed here measures random motility under steady-state conditions, rather than directed migration in response to chemotactic or haptotactic gradients. In the context of myocardial infarction, CF recruitment to the infarct zone is driven by soluble signals, ECM composition, and spatial changes in tissue stiffness. Whether the alterations in random migratory capacity observed following GATA4 deletion reflect a parallel impairment in directed migration remains to be determined. Future studies employing chemotactic gradient assays or transwell migration in the presence of defined growth factor gradients will be necessary to fully evaluate the functional consequences of GATA4 loss on CF recruitment *in vivo*.

Several limitations of this study should be acknowledged. First, the experimental migration studies used neonatal fibroblasts, whereas the public RNA-seq dataset was generated from adult CFs in an independent study. The transcriptomic results therefore provide cardiac-lineage-relevant support but are not a direct molecular readout of the tracked cells. Second, individual tracked cells were derived from a limited number of biological preparations and therefore should not be considered fully independent biological replicates. The cell-level comparisons and Hedges’ g estimates are interpreted as descriptive summaries of the observed distributions. Additional biological replicates and replicate-aware statistical models will be required to confirm the magnitude of these effects. Third, although PDMS substrates reproduce physiological stiffness, they do not reproduce the complete ligand composition, architecture, or spatial heterogeneity of native cardiac ECM. Finally, the RNA-seq analysis identifies associations between *Gata4* loss and cytoskeletal programs but does not establish whether these genes are direct GATA4 targets. Additional studies will be required to define the direct transcriptional mechanisms connecting GATA4 to actin organization, cell polarity, and adhesion dynamics.

Overall, our findings support a model in which GATA4 helps maintain coordination among actomyosin remodeling, cell geometry, and persistent fibroblast migration under physiological matrix stiffness. Loss of GATA4 shifts CFs toward an actomyosin-biased, spread state accompanied by slower and less persistent movement, whereas LFs and TTFs show distinct responses. GATA4 therefore regulates fibroblast migration in a fibroblast-source-dependent manner, with a preferential role in maintaining migratory coordination in cardiac fibroblasts.

## Conclusions

These findings support a model in which GATA4 helps maintain coordination between actomyosin remodeling, cell geometry, and persistent fibroblast migration under physiological matrix stiffness. *Gata4* loss produced an actomyosin-biased, spread cytoskeletal state in CFs that was accompanied by slower and less persistent movement, whereas LFs and TTFs showed distinct responses. GATA4 therefore regulates fibroblast migration in a fibroblast-source-dependent manner, with the most prominent effects observed in CFs under the present experimental conditions.

## Data Availability

The publicly available RNA-seq data analyzed in this study are available in the NCBI Gene Expression Omnibus (GEO) under accession number GSE155358.
